# Biological Valorization of Lignin-Derived Aromatics in Hydrolysate to Protocatechuic Acid by Engineered *Pseudomonas putida* KT2440

**DOI:** 10.3390/molecules29071555

**Published:** 2024-03-30

**Authors:** Xinzhu Jin, Xiaoxia Li, Lihua Zou, Zhaojuan Zheng, Jia Ouyang

**Affiliations:** Jiangsu Co-Innovation Center of Efficient Processing and Utilization of Forest Resources, College of Chemical Engineering, Nanjing Forestry University, Nanjing 210037, China; jxznjfu_1010@163.com (X.J.); fqqdxy2022@163.com (X.L.); lihuazou@njfu.edu.cn (L.Z.); zhengzj@njfu.edu.cn (Z.Z.)

**Keywords:** *Pseudomonas putida* KT2440, sugar loss, protocatechuic acid, biological funnel, lignocellulosic hydrolysate

## Abstract

Alongside fermentable sugars, weak acids, and furan derivatives, lignocellulosic hydrolysates contain non-negligible amounts of lignin-derived aromatic compounds. The biological funnel of lignin offers a new strategy for the “natural” production of protocatechuic acid (PCA). Herein, *Pseudomonas putida* KT2440 was engineered to produce PCA from lignin-derived monomers in hydrolysates by knocking out protocatechuate 3,4-dioxygenase and overexpressing vanillate-*O*-demethylase endogenously, while acetic acid was used for cell growth. The sugar catabolism was further blocked to prevent the loss of fermentable sugar. Using the engineered strain, a total of 253.88 mg/L of PCA was obtained with a yield of 70.85% from corncob hydrolysate 1. The highest titer of 433.72 mg/L of PCA was achieved using corncob hydrolysate 2 without any additional nutrients. This study highlights the potential ability of engineered strains to address the challenges of PCA production from lignocellulosic hydrolysate, providing novel insights into the utilization of hydrolysates.

## 1. Introduction

Lignocellulosic biomass represents a vast and renewable resource for humanity [1]. Its low cost and abundance make it an attractive alternative for the production of fuel and chemicals. Lignocellulosic biomass mainly consists of polysaccharides and lignin [2]. The efficient utilization of both polysaccharides and lignin is critical for the economic efficiency of biomass biorefinery [3]. Over the years, numerous researchers have dedicated their efforts to developing various technologies for the valorization of polysaccharides, including hemicellulose and cellulose [4,5]. The process of separating lignin from a lignocellulosic matrix is well-established and has been used for many years [6,7,8]. However, the utilization of lignin remains challenging in conversion owing to its recalcitrant and diversified structure [9]. Meanwhile, the limited progress in industrial-scale lignin valorization is mainly attributed to the emergence of new products that replace petroleum-derived alternatives but are not cost-effective. To decrease the product cost, there is a growing urgency to develop an efficient method for utilizing waste lignin for the commercialization of lignocellulose biorefinery.

Lignin is the most abundant aromatic feedstock on earth [10]. Every year, approximately 100 million tons of lignin are produced as by-products from various sources [11]. In general, achieving industrial-scale implementation of lignin valorization cannot be accomplished solely through straightforward physicochemical or biological approaches. Combining both of them is a promising solution. Through the physicochemical treatment, a significant amount of monomeric aromatic compounds are released into the pretreated liquor. However, the diverse lignin degradation products are difficult to separate and purify during the chemical upgrading [12]. To prevent the wastage of aromatic resources, some microorganisms are selected and engineered to utilize the monomers to produce high-value-added products. As more and more pathways involved in aromatic metabolism were identified, the biological funnel for the conversion of lignin-related aromatic compound mixtures to bioproducts has been well demonstrated [13,14]. Using alkaline pretreated liquor, Linger et al. achieved 0.252 g/L polyhydroxyalkanoic acid (PHA) by *P. putida* KT2440 [15]. Similarly, 1.8 g/L *cis, cis*-muconic acid was obtained using lignin hydrolysate from softwood as a substrate and recombinant *Corynebacterium glutamicum* MA-2 [16].

Protocatechuic acid (PCA, 3,4-dihydroxybenzoic acid) is a natural catabolic intermediate of lignin and lignin-derived aromatics found in plants and fruits [17]. It has been reported to possess multiple biological activities, such as antioxidation, anti-aging, antibacterial, antiviral, and anti-inflammatory, as reviewed elsewhere [18,19,20]. Given its versatility in the application of pharmaceutical, health food, and cosmetic industries, the production of “natural” PCA has attracted more attention and holds greater business value than that of “artificial” chemical PCA. This is due to its higher price and highly desirable market. However, the preparation of PCA through solvent extraction from plants and fruits often comes with high cost and low yield [21,22]. In recent years, biological approaches for PCA synthesis have been studied using some genetically engineered microorganisms such as *C. glutamicum*, *Saccharomyces cerevisiae*, *Escherichia coli,* and *P. putida*. To date, PCA has been biosynthesized from glucose fermentation through the shikimate pathway and 4.27 g/L and 0.64 g/L of PCA were obtained, respectively, in *E. coli* and *C. glutamicum* [23,24]. Additionally, the highest titer of 12.5 g/L with a yield of 0.313 (g/g) was achieved in *P. putida* KT2440, which implied that *P. putida* KT2440 is an ideal host strain. Apart from shikimate pathways, PCA can also be synthesized from aromatic compounds using the lignin biological funnel at a higher theoretical yield and atom efficiency [16]. Recently, a lignin-derived biosensor in *P. putida* KT2440 was developed for PCA production from a mixture of *p*-coumaric acid (*p*-CA) and ferulic acid (FA) [25]. With vanillin as the substrate, 0.146 g/L of PCA was produced from 0.15 g/L of vanillin by engineered *E. coli* [26]. The biological funnel of lignin offers a new strategy for “natural” PCA production. However, more detailed studies on PCA production using lignin are still limited and need to be further developed.

The hydrolysate derived from lignocellulose following dilute acid pretreatment comprises fermentable sugars and weak acids originating from cellulose and hemicellulose, as well as phenolic compounds derived from lignin. In our previous work, *P. putida* KT2440 exhibited high resistance and tolerance to such hydrolysate [27]. In this work, the degradation abilities of *P. putida* KT2440 for diverse phenolic compounds in hydrolysate were assessed. Based on the biological funnel pathway, an engineered strain capable of producing PCA from phenolic compounds and hydrolysates was constructed. In addition, the genetically modified strain used acetic acid as a carbon source and no longer possessed the capacity to metabolize fermentable sugars, resulting in the retention of sugars in the hydrolysate where they could serve as substrates for other microorganisms to generate novel products.

## 2. Results and Discussion

### 2.1. Evaluation of the Degradation Abilities of P. putida KT2440 towards Phenolic Compounds

**Figure 1:**
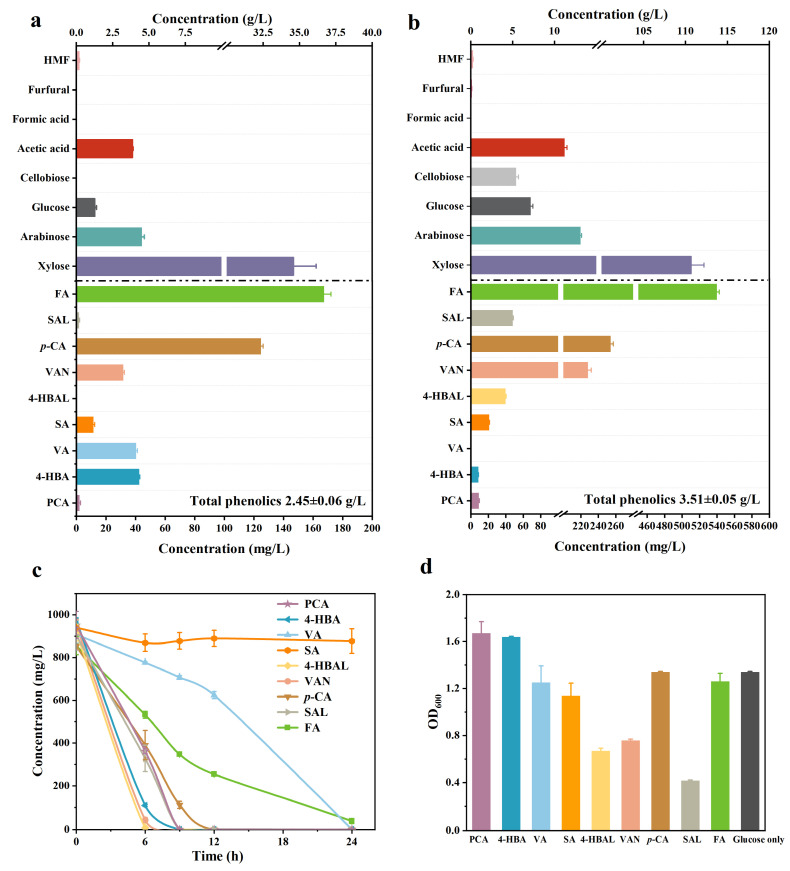
Composition of fermentable sugars, weak acids, furans, and phenolic compounds in (**a**) hydrolysate 1 and (**b**) hydrolysate 2. (**c**) Conversion of 1 g/L of lignin-related monomers present in hydrolysates by KT2440 within 24 h. (**d**) The cell growth profiles of KT2440 after 6 h fermentation in the presence of a simple phenolic compound. Abbreviations: Protocatechuic acid (PCA), ferulic acid (FA), vanillin (VAN), vanillic acid (VA), *p*-coumaric acid (*p*-CA), 4-hydroxybenzaldehyde (4-HBAL), 4-hydroxybenzoic acid (4-HBA), syringaldehyde (SAL), syringic acid (SA). Error bars indicate the standard deviation in two replicates.

Here, two types of corncob hydrolysates obtained from the factory were chosen as representatives for analyzing their composition. Hydrolysate 1 was the liquid fraction directly generated during the dilute acid pretreatment of corncobs, while hydrolysate 2 was obtained by concentrating the liquid fraction after pretreatment using vacuum concentration. As shown in Figure 1a,b, both hydrolysates 1 and 2 are xylose-rich liquors due to degradation of hemicellulose in corncob during pretreatment. Besides xylose, there glucose, arabinose, and cellobiose and their degradation products exist, including weak acids and furans. Their amount is basically consistent with previous similar studies [28]. The phenolic compounds generated from lignin in hydrolysates were at low concentrations of mg/L. Lignin mainly consists of H-type, G-type, and S-type phenyl propane units, which correspond to three different phenolic acids: *p*-CA, FA, and syringic acid (SA) respectively. A variety of low-molecular-weight phenolic compounds, including six phenolic acids and three phenolic aldehydes, was observed in hydrolysates and their proportion of total phenolic is illustrated in Figure S1. Among these, FA and *p*-CA were the main lignin monomers, which account for over 160, 120 mg/L and 530, 250 mg/L, respectively, in hydrolysates 1 and 2.

In order to evaluate the bio-degradation ability of *P. putida* KT2440 for phenolic compounds in corncob hydrolysate, the above-determined lignin degradation products were individually applied to fermentation by *P. putida* KT2440. The concentration of phenolics was 1 g/L, and 10 mM glucose was added to the M9 minimal medium for keeping the cell growth. As illustrated in Figure 1c, *P. putida* KT2440 degraded most phenolics within 24 h except for SA. Despite *P. putida* KT2440 did not consume SA, it was seen that glucose in the medium was completely used up (Figure S2a) and the cell still showed significant growth (Figure 1d). This implied that *P. putida* KT2440 lacks the degradation metabolism for SA, but still demonstrates good tolerance for SA. Compared with phenolic acids, the results of cell growth in Figure 1d showed that phenolic aldehydes exhibit higher toxicity than phenolic acids for *P. putida* KT2440. Among three phenolic aldehydes, syringaldehyde (SAL) has the strongest toxicity, followed by vanillin (VAN) and 4-hydroxybenzaldehyde (4-HBAL). After 24 h fermentation, all of these phenolic aldehydes were found to be converted to the corresponding phenolic acids through aldehyde oxidation reactions [29]. As shown in Figure S2b,c, *P. putida* KT2440 could convert SAL into SA, which was accumulated due to lack of further degradation pathway during fermentation, while the amount of VA was observed in the first 36 h and completely consumed at the end during fermentation of VAN. Moreover, VA also served as an intermediate product during FA degradation (Figure S2d). This suggests that VA is located downstream point of FA and VAN pathways and that the production of VA is faster than its consumption [30].

Overall, *P. putida* KT2440 demonstrated good abilities for the degradation of a variety of low-weight phenolic compounds at 1 g/L. All of the H-type lignin-derived compounds, such as *p*-CA, 4-HBAL, and 4-HBA, are quickly consumed within 12 h. In comparison, VAN, VA, and FA from G-type lignin exhibited a relatively slower degradation rate. Even so, a complete degradation also occurred within a 24 h period. These results suggest that *P. putida* KT2440 is capable of efficiently utilizing the majority of aromatic monomers in hydrolysates. Hence, it is a suitable starting strain for efficiently converting aromatic monomers derived from lignin into products.

### 2.2. Effects of Sugar and Weak Acid in Hydrolysates on Phenolic Compound Metabolism

**Figure 2:**
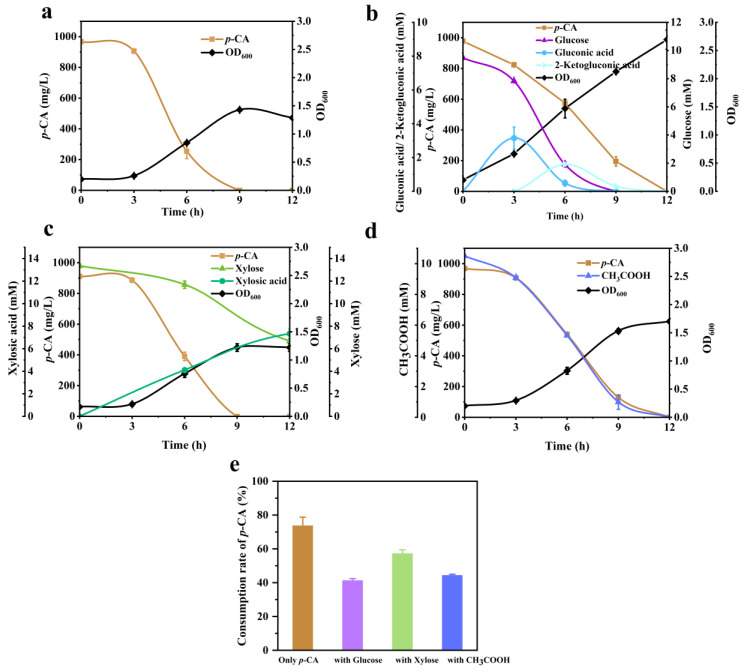
Fermentation profiles of KT2440 with additional supplement of different carbon sources in the presence of *p*-CA. (**a**) *p*-CA only, (**b**) 10 mM glucose, (**c**) 10 mM xylose, and (**d**) 10 mM acetic acid in 12 h. The consumption rate of *p*-CA at 6 h under four environments was shown by (**e**). Error bars indicate the standard deviation in two replicates.

According to the component analysis of hydrolysates, there are many other chemicals that existed in the liquor, mainly sugar and weak acids except for phenolic compounds. The metabolism by which these compounds interfere with the degradation of phenolic compounds is unknown when using *P. putida* KT2440. Thus, Figure 2 shows the fermentation performance of *P. putida* KT2440 using sugar and a weak acid, either the sole or the mixture.

When using *p*-CA as the sole carbon source, *P. putida* KT2440 completely metabolized *p*-CA within 9 h and there was no obvious product accumulated. Meanwhile, significant cell growth was observed in the first 9 h. It suggested that *P. putida* KT2440 could directly use *p*-CA as the sole carbon source for cell growth. As supplemented with 10 mM glucose, *P. putida* KT2440 promoted better cell growth throughout the process than that of *p*-CA as the sole carbon source. However, glucose supplementation delayed *p*-CA metabolism to 12 h (Figure 2b). Both glucose and *p*-CA were used up within 12 h. The accumulation of gluconic acid and 2-ketogluconic acid was observed momently at 3 h and 6 h, respectively. Previously, it was reported that glucose is first oxidized to gluconate or 2-ketogluconic acid, then transported to the cytosol and finally enters the TCA cycle in *P. putida* KT2440 [31]. Hence, glucose is proposed as the preferred carbon and energy source over *p*-CA, and the presence of glucose inhibits the metabolism of *p*-CA. In Figure 2c, the conversion of xylose was lagged behind *p*-CA, but it did not have a significant effect on cell growth. The consumption of xylose was basically consistent with the production of xylonic acid. *p*-CA is still the main carbon and energy source and xylose is mainly oxidized to xylonic acid [32]. Acetic acid is a kind of typical byproduct of hydrolysate. In *P. putida* KT2440, acetic acid was consumed within 12 h (Figure 2d), indicating that acetic acid could serve as a potential carbon source.

Figure 2e summarizes the consumption rate of *p*-CA at 6 h fermentation by *P. putida* KT2440 using four different types of carbon sources. It was found that the presence of glucose, xylose, and acetic acid all slowed down the degradation rate of *p*-CA. The consumption rates decreased to 41.19%, 57.12%, and 44.23%, respectively, lower than the control rate of 73.62%. When hydrolysate was directly used for the bioconversion of lignin-derived compounds, this inhibition effect would be a major obstacle. However, it should be noted that once *p*-CA is designed to be converted into one product by engineering *P. putida* KT2440, its effect as a carbon and energy supplier needs to be weakened due to its main flow distribution towards conversion. By that time, glucose and acetic acid in hydrolysates would be used as alternative carbon and energy sources for cell growth. Furthermore, considering the significant inhibition of *p*-CA metabolism in *P. putida* KT2440 by glucose, acetic acid appears to be a suitable and effective carbon source for maintaining cell activation in real hydrolysate applications.

### 2.3. Construction of an Engineered P. putida Strain for Protocatechuic Acid Production

**Figure 3:**
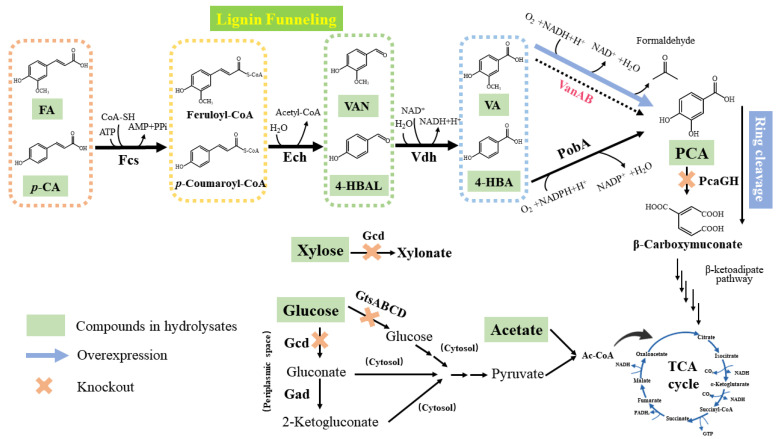
Metabolic pathways of substances present in hydrolysate in *P. putida* KT2440 with engineered pathways are shown. Abbreviations: *p*-coumarate-CoA ligase (Fcs), enoyl-CoA hydratase/aldolase (Ech), vanillin dehydrogenase (Vdh), *p*-hydroxybenzoate hydroxylase (PobA), vanillic acid O-demethylase oxygenase (VanAB), glucose dehydrogenase (Gcd), glucose ABC transporter (GtsABCD), gluconate dehydrogenase (Gad), tricarboxylic acid cycle (TCA cycle).

Preliminary tests indicated that *P. putida* KT2440 was capable of efficiently utilizing most aromatic monomers commonly found in hydrolysates; however, it was unable to produce any valuable product. In general, the majority of aromatic compounds undergo complete degradation via biological funneling and the β-ketoadipate pathway before entering the TCA cycle [33]. Biological funneling is the process by which various aromatic compounds in hydrolysates converge to PCA which serves as their common intermediate metabolite. As mentioned in Figure 3, all six phenolic compounds in hydrolysates could be converted into PCA and then split by *pcaGH*. Specifically, these phenolic compounds can be divided into two metabolic pathways: G-lignin-derived compounds (FA, VAN, and VA) are funneled into VA and further oxidized to PCA by vanillic acid O-demethylase oxygenase (VanAB); *p*-CA, 4-HBAL, and 4-HBA from H-lignin-derived compounds are funneled into 4-HBA and further degraded to PCA by *p*-hydroxybenzoate hydroxylase (PobA). Regardless of their origin, it has been found that knocking out of the gene *pcaGH* is necessary for the accumulation of PCA.

**Figure 4:**
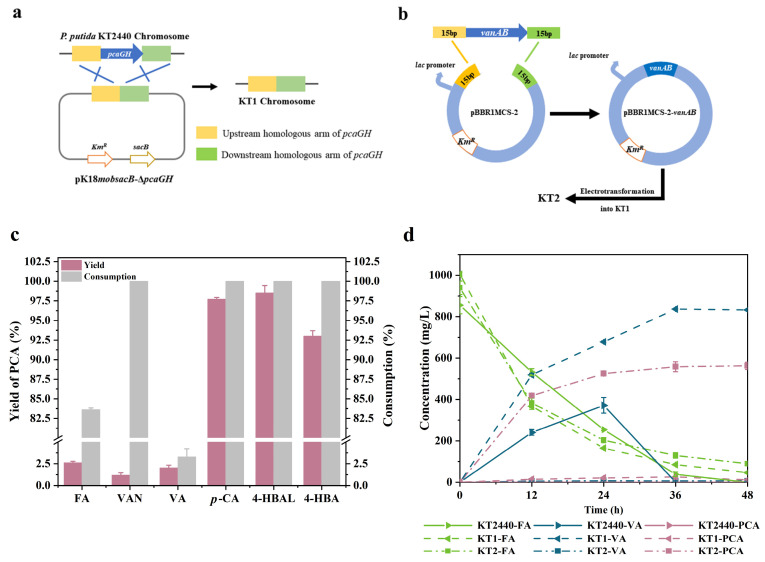
Construction of PCA-producing strains and removal of restriction factors for PCA production. (**a**) Overview of *pcaGH* knockout by pK18*mobsacB* and (**b**) *vanAB* overexpression by pBBR1MCS-2, (**c**) Histogram of PCA yield and consumption of monophenols by KT01. (**d**) Shake-flask evaluation of the effect of knockout and overexpression on PCA production from FA by KT2440, KT1, and KT2. Error bars indicate the standard deviation in two replicates.

By a homologous recombination knockout technology [27] shown in Figure 4b, the engineered strain KT1 was obtained by knockout of *pcaGH* (Figure S3). Using the KT1, we investigated the production of PCA using six different phenolic compounds at a concentration of 1 g/L supplemented with 10 mM glucose as a carbon source. As shown in Figure 4c, the knockout of *pcaGH* did not significantly influence the consumption of phenolic compounds, with the majority being used within 24 h except for FA and VA. however, only three H-lignin-derived monomers, *p*-CA, 4-HBAL, and 4-HBA, demonstrated exceptional abilities for PCA production, whose yields of PCA were 97.7%, 98.5%, and 93.1%, respectively. The complete conversion and production demonstrated the uninterrupted flow of this pathway to PCA. Meanwhile, the block of the PCA to TCA cycle did not exert any discernible impact on cellular proliferation. One plausible explanation is that the presence of glucose potentially furnishes an abundant supply of carbon and energy. In a previous study, the knockout of *pcaGH* in the KT2440 strain resulted in a production of only 5.2 mg/L of PCA from glucose [34]. Our results suggested that the production of PCA from *p*-CA may be more efficient compared to glucose due to a shorter metabolic pathway and higher atom efficiency.

Regarding H-lignin-derived monomers, the consumptions of FA, VAN, and VA were 83.7%, 100%, and 3.3% after 24 h fermentation. However, all yields of PCA were less than 3% (Figure 4c). Meanwhile, a large amount of VA was produced or maintained during the fermentation process (Figure 4d). The result suggested that the oxidation of VA into PCA by the *vanAB* encoding enzymes was seriously inhibited owing to the block out of the downstream pathway. Thus, it was speculated that the expression of the *vanAB* gene in the *P. putida* KT2440 genome was regulated by the downstream pathway. Therefore, to enhance the expression of *vanAB*, the endogenous *vanAB* gene was introduced into KT1 by overexpression, generating KT2. pBBR1MCS-2 containing a lac promoter was used as the expression vector to overexpress *vanAB.* Endogenous *vanAB* was ever reported to be a key rate-limiting step in the FA pathway [34]. Upadhyay and Lali overexpressed several different sources of *vanAB* genes in KT2440 ∆*pcaGH* and found that the conversion rate of VA to PCA could increase to 51.33% from 10.54% by overexpressing *vanAB* from *Acinetobacter* sp. ADP1 [35]. In this work, both KT1 and KT2 were able to completely consume 1 g/L of FA almost totally within 36 h, but the overexpression of the *vanAB* gene in KT1 led to a significant increase in PCA yield from 2.61% to 75.63%, while only a minimal accumulation of VA was observed (Figure 4d). The construction of an engineered strain of *P. putida* KT2440 capable of efficiently producing PCA from *p*-CA and FA has been successfully accomplished so far.

### 2.4. PCA Production from Single Phenolic Acid in the Engineered P. putida

**Figure 5:**
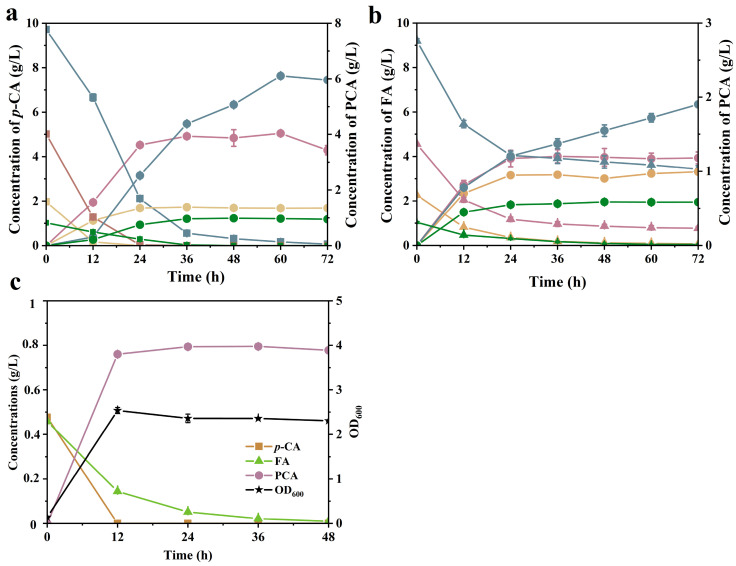
Assessment of PCA production by KT2 in the presence of glucose using different concentrations of (**a**) *p*-CA and (**b**) FA and their production of PCA. (**c**) PCA production using mixed phenolic acid (*p*-CA:FA = 1:1) by KT2 in the presence of glucose. Squares represent *p*-CA, circles represent PCA, and triangles represent FA. The blue line corresponds to 10 g/L substrate concentration, the pink line corresponds to 5 G/L, the yellow line corresponds to 2 g/L, and the green line corresponds to 1 g/L. Error bars indicate the standard deviation in two replicates.

Firstly, the production boundaries of PCA by KT2 were investigated using *p*-CA and FA at three different concentration levels (1, 2, 5, and 10 g/L). Figure 5a shows a complete conversion of 10 g/L *p*-CA within 72 h, resulting in a maximum PCA production of 6.11 g/L. Despite the PCA production from *p*-CA being far from that from glucose [24], its high yield by KT2 confirmed its potential to form lignin into PCA. In terms of the production of PCA from FA (Figure 5b), the assimilation of FA exhibited significant delays compared to the *p*-CA pathway. Only 2.5 g/L of FA could be consumed within 72 h and the maximum concentration of PCA was at only 1.9 g/L. This comparison suggested that further enhancements were required for efficient PCA production from FA in the future.

A comparison of the PCA production pathways from *p*-CA and FA revealed a shared partial pathway (Figure 3). Their difference in degradation ability was mainly dependent on the types of substrates, implying that *p*-CA utilization was preferred. As the substrate loading increased, the PCA yield decreased whether for *p*-CA or for FA. At a high substrate loading (10 g/L), the PCA yield from *p*-CA and FA decreased to 67.0% and 22%, respectively, accompanied by the accumulation of the intermediate products of 4-HBA and VA (Figure S4a). The accumulation of intermediate products indicates an inadequate conversion in the final step of PCA production. For *p*-CA and FA, *pobA* and *vanAB* were, respectively, responsible for the PCA biosynthesis from 4-HBA and VA, which required distinct cofactors, NADPH and NADH. By comparison, the accumulation of VA was more serious than 4-HBA (Figure S4b). The decrease in PCA yield might be attributed to the inadequate activity of *pobA* with increasing concentration of *p*-CA. For FA metabolism, it might be due to an excess unbalance of redox metabolism [36]. Additionally, the detrimental impact of formaldehyde as a by-product should also be taken into account due to its inherent toxicity.

### 2.5. PCA Production from Hydrolysates

As mentioned above, although the PCA yield decreased with increasing substrate loading, KT2 was still capable of meeting the demand for biosynthesis of PCA from lignin hydrolysates due to the presence of a low concentration of phenolic acid. However, several factors still influenced the conversion. A series of aromatics, mainly including *p*-CA, FA, and their related phenolic compounds, coexist in the hydrolysates (Figure 1). Hence it was necessary to assess the co-utilization efficiency of *p*-CA and FA. In Figure 5c, a mixture of *p*-CA and FA in a 1:1 (*w/w*) ratio was used at a total of 1 g/L loading in the presence of 10 mM glucose. The strain KT2 depleted the two phenolic acids simultaneously, achieving a maximum yield of 98.1% corresponding to 0.8 g/L PCA.

Besides phenolic compounds, the hydrolysates also contained sugars and acetic acids. Previous research indicated that the presence of glucose contradicted the mechanism of phenolic compound degradation due to the existence of a global regulator (crc), whose deletion promoted the expression of *pobA* and *vanAB* [37]. Additionally, xylose was present at a relatively high level in two hydrolysates. Although xylose has little influence on *p*-CA metabolism and cell growth, its consumption would result in a massive accumulation of xylonic acid, which would affect the separation of the product during the downstream process. In light of the aforementioned drawbacks of sugar metabolism, it was imperative to further impede the sugar utilization pathway. As an alternative approach, harnessing acetic acid from hydrolysates as a carbon source held promise for sustaining cell growth.

**Figure 6:**
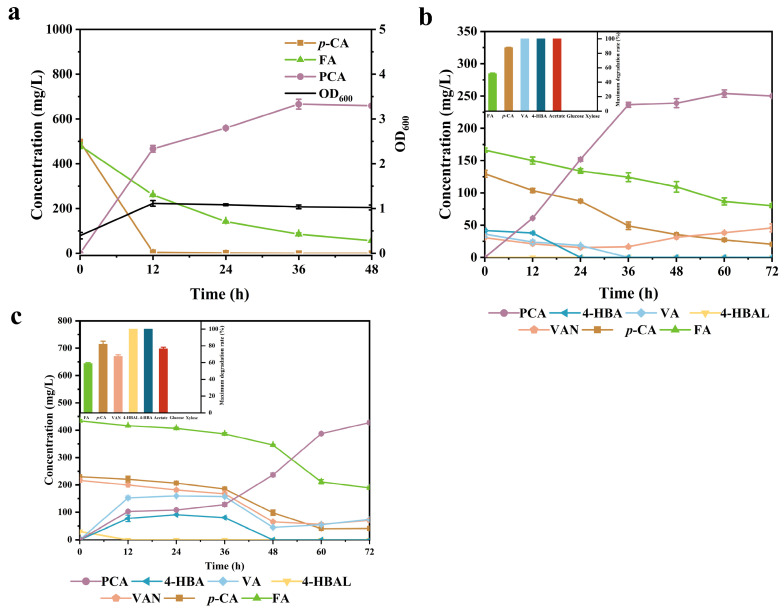
Production of PCA from mixed phenolic acid and real hydrolysates by KT3. (**a**) PCA production using mixed phenolic acid (*p*-CA and FA) in the presence of acetic acid. The fermentation of KT3 for PCA production and the maximum degradation rate of each substance from (**b**) hydrolysate 1 and (**c**) 80% hydrolysate 2. Error bars indicate the standard deviation in two replicates.

As mentioned in Section 2.2, the genes *gcd* and *gtsABCD* were knockout and the strain KT3 was obtained based on KT2. Due to the lack of these genes, KT3 lost the ability to catabolize xylose and glucose for cell growth (Figure S5). When using 1 g/L acetic acid as an additional carbon source instead of glucose, the PCA-producing ability of KT3 remained largely unaffected by the knockout of *gcd* and *gtsABCD* (Figure 6a). The strain KT3 synchronously converted 94.2% of the two phenolic acids with a maximum PCA yield of 78.4%, equivalent to 0.6 g/L PCA. There was no significant intermediate metabolite accumulation during the whole fermentation process. Meanwhile, 2.75-fold cell growth was observed within 24 h. Furthermore, two hydrolysates as complex mixtures were used to investigate the effect on KT3. Interestingly. KT3 demonstrated a robust performance for a real, heterogeneous, lignin-enriched, hydrolysate-derived pilot-scale biomass pretreatment. Furthermore, 100% of hydrolysate 1 could be mechanized well without any dilution or other additional substances. Meanwhile, 253.88 mg/L PCA was achieved from hydrolysate1 with a yield of 70.85% (Figure 6b). The value of yield was nearly the same as that of mixed phenolic acid (Figure 5a). The amount of all five phenolic compounds in hydrolysate 1 decreased by a maximum of 52–100%, indicating that they were converted into PCA through the bio-funneling pathway. Among them, FA degradation was the worst one with only 52%. This outcome was likely attributed to the limited efficiency of the FA metabolism in *P. putida*. Hydrolysate 2 exhibited more serious toxicity than hydrolysate 1, probably due to the presence of a higher concentration of phenolic acid. Since KT3 could not survive in hydrolysate 2, a diluted solution of hydrolysate 2 mixed with sterilized water at an 80% concentration was used for PCA production. As shown in Figure 6c, using 80% hydrolysate 2, the optimal production of 433.72 mg/L PCA was observed by KT3 with a yield of 56.74%. 4-HBA as intermediates were observed shortly and completely depleted at the end. All glucose and xylose were retained, and the residual acetic acid concentration of 1.9 g/L corresponded to a degradation rate of 76.31%. However, KT2 only produced 111.77 mg/L of PCA, corresponding to a yield of 11.34%. Additionally, not only FA but also the metabolic process of 4-HBA was suppressed, highlighting the significance of disrupting the *gcd* and *gtsABCD* genes (Figure S6).

Table 1 summarizes the production of PCA from aromatic compounds and lignocellulose hydrolysate. Previous studies were mainly limited to the conversion of two phenolic acids (*p*-CA and FA). Hydrolysate is a complex system, which inevitably contains a large amount of xylose and weak acid. The utilization of hydrolysate in the past has relied on sugar as a carbon source, resulting in increased sugar consumption and the production of xylonic acid as a by-product. For the first time, acetic acid was used as the carbon source for the transformation, and the constructed strain could obtain a single product by co-conversion of multiple substances at the same time. Furthermore, the absence of sugar loss makes it suitable for subsequent biorefining processes.

## 3. Materials and Methods

### 3.1. Materials

Corncob was composed of 34.5% cellulose, 30.9% hemicellulose, and 22.7% lignin. After pretreatment with dilute sulfuric acid at 160 °C for 60 min, the remaining solid residue was composed of 54.6% cellulose, 3.1% hemicellulose, and 32.3% lignin. The hydrolysate pretreatment was kindly provided by a biomass factory in China. Syringaldehyde and ferulic acid were purchased from TCI (Shiga, Japan). 4-hydroxybenzoic acid, vanillic acid, syringic acid, 4-hydroxybenzaldehyde, vanillin, and *p*-coumaric acid were purchased from Sigma-Aldrich (Saint Louis, MO, USA).

### 3.2. Strains and Plasmids

All bacterial strains and plasmids used in this study are listed in Table 2. *E. coli Trans*1-T1 was purchased from TransGen (Beijing, China). *P. putida* KT2440 was obtained from the Biochemical Engineering Research Institute of Nanjing Forestry University. *P. putida* KT1, KT2, and KT3 were independently constructed in this study.

### 3.3. Genetic Manipulation and Plasmid Construction

The genome of *P. putida* KT2440 was extracted by TaKaRa MiniBEST Bacterial Genomic DNA Extraction Kit Ver.3.0 (Takara, Shiga, Japan).

As for the construction of a *pcaGH* (PP_4655-4656) disruption mutant, all procedures were conducted using the protocol previously [27]. Briefly, the 500 bp upstream and downstream homology arms of *pcaGH* were amplified from the *P. putida* KT2440 genome by primers *pcaGH*-up-f/r and *pcaGH*-down-f/r respectively. After gel purification by Wizard^®^ SV Gel and PCR Clean-Up System (Promega, Wisconsin, USA), overlap extension PCR was performed, using primers *pcaGH*-up-f and *pcaGH*-down-r and thus the upstream and downstream regions were fused. The purified 1000 bp PCR products were then ligated with the pEASY-Blunt cloning vector to form a new plasmid. The plasmid and pK18*mobsacB* were digested using *Eco*R I and *Bam*H Ⅰ to generate cohesive ends and then ligated to obtain *pcaGH* knockout plasmid code as pK18*mobsacB*-Δ*pcaGH*. The plasmid was introduced into *P. putida* KT2440 by electro-transformation. Single crossovers in which the plasmid had recombined into the genome were selected on LB plates supplemented with 50 μg/mL kanamycin. After two rounds of passaging in LB broth with 15% (*w/v*) sucrose without antibiotics, the correct double-crossover transformants were selected using LB agar plates supplemented with 15% (*w/v*) sucrose, referred to as KT1. The deletion was found successful on the basis of the shortened PCR product of 1000 bp using the primers *pcaGH*-up-f and *pcaGH*-down-r (Figure S3). KT1 was used as the starting strain for further construction of *gcd* and *gtsABCD* knockout mutant generating KT1 Δ*gcd*Δ*gtsABCD*. The process of knockout was the same as described above.

Regarding the overexpression of the gene *vanAB* (PP_4655-4656) from *P. putida* KT2440, pBBR1MCS-2 was used to express *vanAB*. The gene *vanAB* was amplified from *P. putida* KT2440 genome with primers *vanAB*-f/r. Then using pBBR1MCS-2-f/r to obtain a linearized vector. At last, the target gene was recombined with the linearized vector using the pEASY^®^-Basic Seamless Cloning and Assembly Kit (TransGen Biotech Co., Ltd., Beijing, China), generating plasmid pBBR1MCS-2-*vanAB*. The plasmid was electroporated into KT1 and KT1 Δ*gcd*Δ*gtsABCD*, after screening single colonies on LB agar plates with 50 μg/mL kanamycin, the plasmid extraction was verified and the correct strains were marked as KT2 and KT3.

### 3.4. P. putida Cultivations

*P. putida* KT2440 and its derived strains were all pre-cultured overnight in 5 mL Luria-Bertani (LB) medium (10 g/L tryptone, 5 g/L yeast extract, 10 g/L NaCl) at 30 ℃ and 200 rpm. If required, 50 μg/mL kanamycin was added to the medium to avoid loss of plasmid.

For the fermentation of lignin-derived monomers, the overnight cultures were harvested by centrifugation at 8000 rpm for 5 min, after discarding the supernatant, the cells were resuspended twice with 0.9 (*w/v*) NaCl. The initial OD_600_ was set to 0.2 in a 250 mL shake flask containing 10% filling volume of M9 minimal medium (2 mM MgSO_4_, 0.1 mM CaCl_2_, M9 salts (12.8 g/L Na_2_HPO_4_·7H_2_O, 3 g/L KH_2_PO_4_, 0.5 g/L NaCl, 1.0 g/L NH_4_Cl), trace element solution (0.3 g/L H_3_BO_3_, 0.05 g/L ZnCl_2_, 0.03 g/L MnCl_2_·4H_2_O, 0.2 g/L CoCl_2_, 0.01 g/L CuCl_2_·2H_2_O, 0.02 g/L NiCl_2_·6H_2_O, 0.03 g/L NaMoO_4_·2H_2_O) then the volume was made up to 1 L with distilled water) supplemented with carbon sources and aromatic compounds. The pH of the medium was adjusted by 10 M NaOH. 10 mM glucose was the carbon source for *P. putida* KT2440, KT1, and KT2, while KT3 utilized 16 mM acetate as its carbon source.

For the fermentation of the hydrolysates, CaO was used to adjust the pH of the hydrolysate to 7.2. KT3 was pre-activated overnight, and then 1% seed cultures were inoculated into 25 mL of LB medium in a 250 mL shake flask for 12 h. The initial OD_600_ was set to 5 in a 100 mL shake flask containing 10% filling volume of hydrolysates. Hydrolysate 2 was diluted with sterilized distilled water. All fermentations were carried out at 200 rpm and 30 °C. All samples were centrifuged at 12,000 rpm for 5 min. The supernatant was diluted to the appropriate concentration using deionized water in preparation for further analysis.

### 3.5. Analytical Methods

The consumption of glucose and acetate acid was detected by an Agilent 1260 HPLC system equipped with an Aminex HPX-87H column (Bio-Rad, Hercules, CA, USA) which had a refractive index, the above components were separated at 55℃ using 5 mM H_2_SO_4_ as eluent at a flow rate of 0.6 mL/min [28].

The concentrations of phenolic compounds were identified by using an HPLC system with a Zorbax SB-C18 column (150 × 4.6 mm, Alto, CA, USA) with a UV detector. The gradient elution was performed using 100% acetonitrile as mobile phase A and 1.5% acetic acid as mobile phase B at a flow rate of 0.8 mL/min [40].

Xylose, xylonic acid, gluconate, and 2-ketogluconate were analyzed by high-performance anion exchange chromatography (Thermo, ICS-3000, Waltham, MA, USA) equipped with a CarboPac™ PA10 column and pulsed amperometric detection set at 30 °C. The mobile phase was 100 mM NaOH and 500 mM NaAc at a flow rate of 0.3 mL/min [41]. All experiments were performed at least in duplicate, and values were expressed as mean ± standard deviation.

Cell growth was monitored turbidimetrically at an optical density of 600 nm (OD_600_).

The yield of PCA is calculated as the ratio of PCA (mol) to the sum of the initial monophenols (mol).

## 4. Conclusions

In this work, *P. putida* KT2440 exhibited remarkable degradation abilities toward diverse phenolic acids and aldehydes in hydrolysates. By knockout of *pcaGH* and overexpression of the endogenous *vanAB* in *P. putida* KT2440, the maximum yield of PCA from FA and *p*-CA increased to 75.6% and 97.7%, respectively. Furthermore, by blocking sugar metabolism, the engineered strain KT3 did not consume the sugar, and only utilized the residual acetic acid in hydrolysates for cell growth and converted at least five phenolic compounds to PCA. Additionally, 253.88 mg/L of PCA was achieved by hydrolysate 1 with a yield of 70.85% and the highest titer of PCA was obtained at 433.72 mg/L PCA using hydrolysate 2. In addition to the common chemicals from sugars, the production of PCA further enhances the utilization value of the dilute acid pretreatment hydrolysate.

## Figures and Tables

**Table 1:** Comparative PCA production reported from aromatic compounds and lignocellulose hydrolysate.

Strain	Substrate	Fermentation Duration (h)	Titer (g/L)	Yield	Reference
Engineered *P. putida* KT2440	1 g/L VA	n. a.	0.51	0.51	[35]
*P. putida* KT14	3.28 g/L *p*-CA	48	3.1	1.00	[25]
*S. Cerevisiae* yPCA12	1.6 g/L *p*-CA	96	0.72	0.48	[38]
*C. glutamicum* ATCC 21420 FVan	0.89 g/L FA	48	0.44	0.63	[39]
*P. putida* KT2440 KT16	3.88 g/L FA	48	2.1	0.68	[25]
*P. putida* KT2	1.02 g/L *p*-CA	72	0.95	0.99	This work
*P. putida* KT2	1 g/L FA	72	0.76	0.76	This work
*P. putida* KT2	*p*-CA:FA = 1:1 (mol/mol)	48	0.8	0.98	This work
*S. cerevisiae* yPCA12	0.5×Corn stover APL	96	0.81	0.74 ^a^	[38]
*P. putida* KT3	Corncob hydrolysate 1	72	0.25	0.70 ^b^	This work
*P. putida* KT3	Corncob hydrolysate 2	72	0.43	0.57 ^b^	This work

^a^ PCA yield was based on the total mol of FA and *p*-CA; ^b^ PCA yield was based on the total mol of FA, *p*-CA, VAN, 4-HBAL, VA, and 4-HBA; n. a.: not available; APL: alkaline pretreatment liquor.

**Table 2:** Strains and plasmids used in this study.

Strains/Plasmids	Characteristics	Source
*Escherichia coli*		
*Tans*1-T1	Cloning host	TransGen Biotech
*Pseudomonas putida*		
KT2440	Wild-type strain	Lab stock
KT1	KT2440 with scarless deletion of *pcaGH* (PP_4655-4656) encoding protocatechuate 3,4-dioxygenase dehydrogenase	This study
KT2	KT01 harboring plasmid pBBR1MCS2-*vanAB* (PP_3736-3737) encoding vanillate O-demethylase oxygenase	This study
KT3	KT02 with scarless deletion of glucose dehydrogenase encoding *gcd* (PP_1444) and glucose ABC transporter *gtsABCD* (PP_1015-1018)	This study
Plasmids		
pBBR1MCS2	Broad-host-range cloning vector; Km^R^	Lab stock
pK18*mobsacB*	The suicide vector containing the *sacB* gene; Km^R^	Lab stock
pBBR1MCS2-*vanAB*	pBBR1MCS2 with *vanAB* from *P. putida* KT2440	This study
pK18*mobsacB*-Δ*pcaGH*	pK18*mobsacB* containing the homology arms of *pcaGH* from *P. putida* KT2440	This study

## Data Availability

The data presented in this study are available on request from the corresponding author.

## Supplementary Materials

The following supporting information can be downloaded at: https://www.mdpi.com/article/10.3390/molecules29071555/s1, Figure S1: Proportion of nine monophenols in total phenols in two hydrolysates; Figure S2: Metabolic process and growth of SAL, SA, VAN, and FA by *P. putida* KT2440; Figure S3: Confirmation of gene deletion and overexpression; Figure S4: The intermediate concentration (4-HBA and VA) curves; Figure S5: The growth on glucose and xylose by engineered *P. putida* KT2440; Figure S6: Production of PCA from 80% (*v/v*) hydrolysate 2 by KT2; Figure S7: HPLC analysis of PCA production in hydrolysates 1 and 2. Table S1: Primers used in this study.
