# Supplementary material for: Biological Valorization of Lignin-Derived Aromatics in Hydrolysate to Protocatechuic Acid by Engineered Pseudomonas putida KT2440
Source: Molecules. 2024 Mar 30;29(7):1555. doi: 10.3390/molecules29071555 (PMC11013400; doi:10.3390/molecules29071555)
Supplement: Supplementary file 1 [file molecules-29-01555-s001.zip › molecules-2895092-supplementary.pdf]

Supplementary Materials

Biological valorization of lignin-derived aromatics in hydrolysate to protocatechuic acid by engineered *Pseudomonas putida* KT2440

Xinzhu Jin[1], Xiaoxia Li[1], Lihua Zou[1], Zhaojuan Zheng[1], Jia Ouyang[1*]

[1]Jiangsu Co-Innovation Center of Efficient Processing and Utilization of Forest Resources, Nanjing Forestry University, Nanjing 210037, People’s Republic of China;

Corresponding author: College of Chemical Engineering, Nanjing Forestry University, No. 159 Longpan Road, Nanjing 210037, People’s Republic of China.

Tel/Fax: +86-02585427129.

E-mail address: hgouyj@njfu.edu.cn (J. Ouyang)

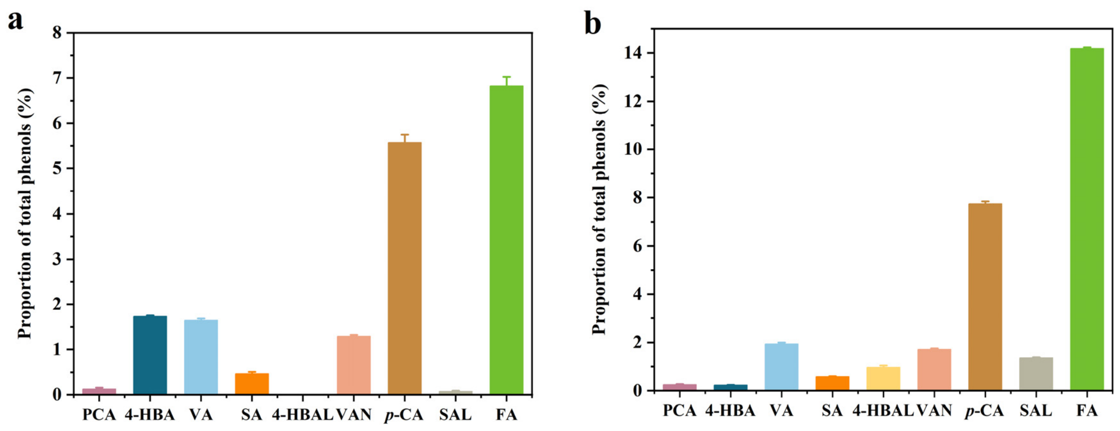

Fig. S1 Proportion of nine monophenols in total phenols in (a) hydrolysate 1 and (b) hydrolysate 2.

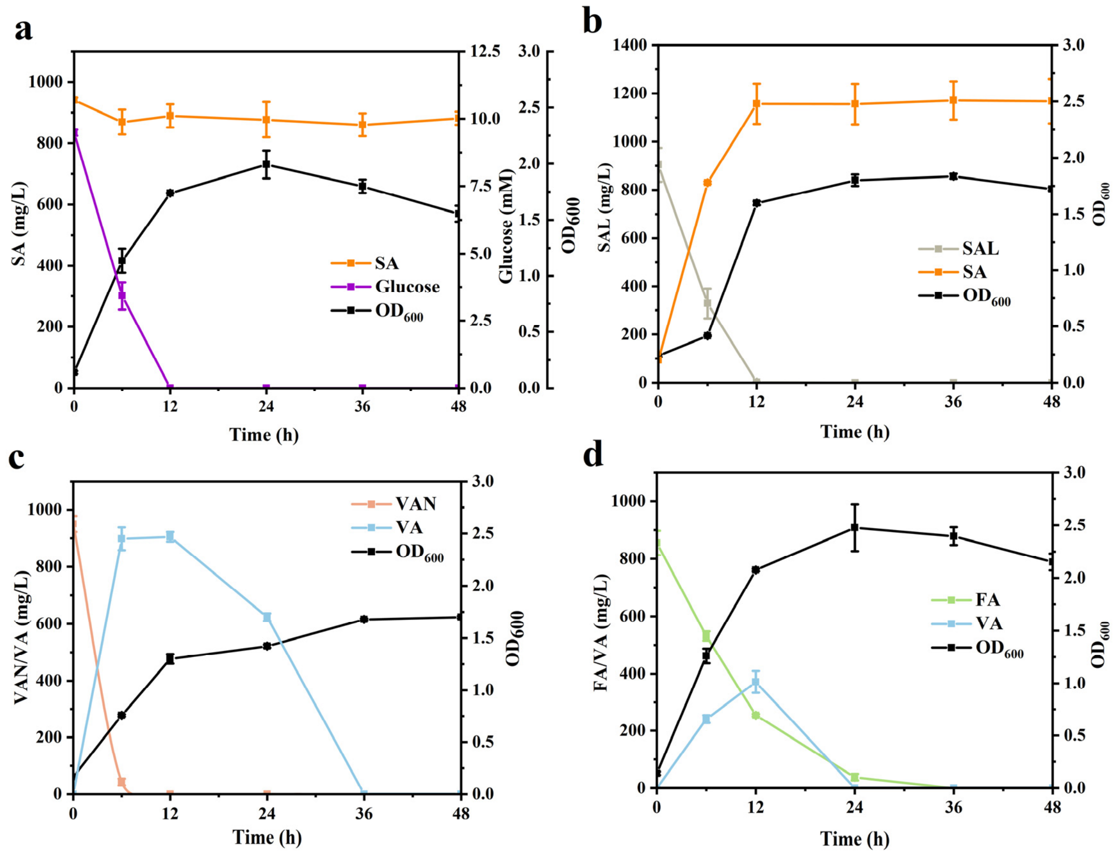

Fig. S2 Metabolic process and growth of (a) syringaldehyde, (b) syringic acid, (c) vanillin, (d) ferulic acid (1 g/L) by KT2440 in M9 minimal medium with 10 mM glucose (OD_{600}=0.2).

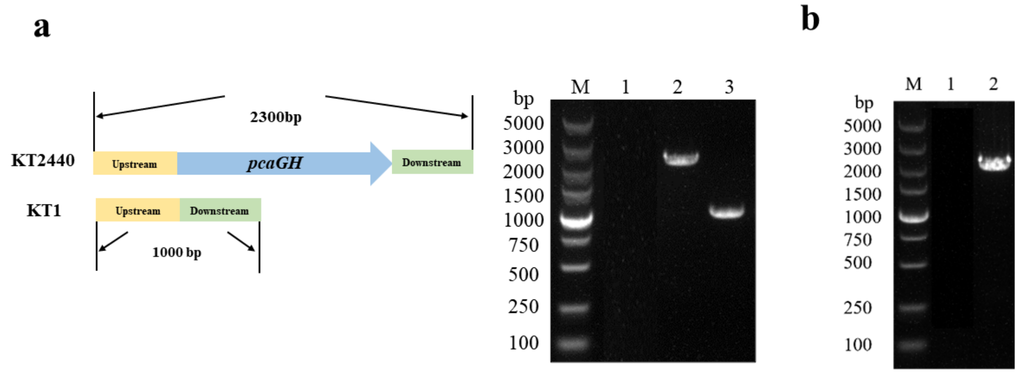

Fig. S3 (a) Confirmation of the deletion of the *pcaGH* gene in *P. putida* KT2440 genome, using colony PCR. The primers *pcaGH*-up-f and *pcaGH*-down-r were used for PCR. The positive clone exhibited a 1000 bp fragment that corresponded to the expected size of the deletion. M, 5 kb DNA ladder; 1, blank; 2, *P. putida* KT2440; 3, KT1. (b) Confirmation of the overexpression of the *vanAB* gene in KT1, using PCR after extraction of the plasmid, the correct 2031 bp fragment was shown. M, 5 kb DNA ladder; 1, blank; 2, KT2.

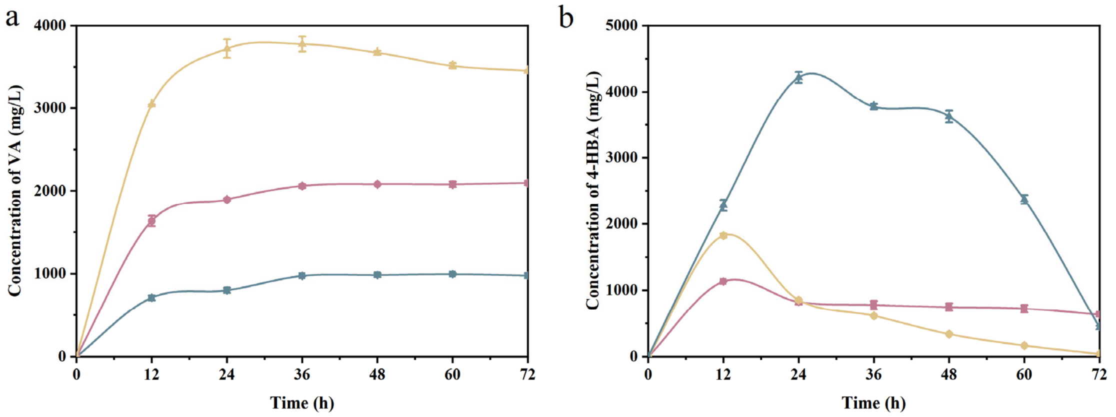

Fig. S4 (a) The intermediate 4-HBA concentration curves under different concentrations of *p*-CA. (b) The intermediate VA concentration curves under different concentrations of FA. The blue line corresponds to 10 g/L substrate concentration, the yellow line corresponds to 5 g/L, the pink line corresponds to 2 g/L, and the green line corresponds to 1 g/L.

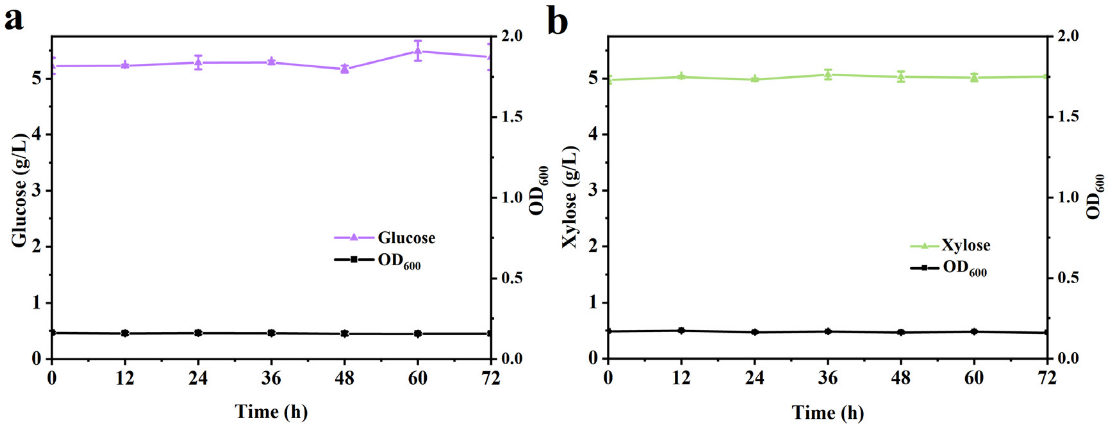

Fig. S5 Growth characterization of KT3 in M9 minimal medium with (a) 5 g/L glucose and (b) 5 g/L xylose.

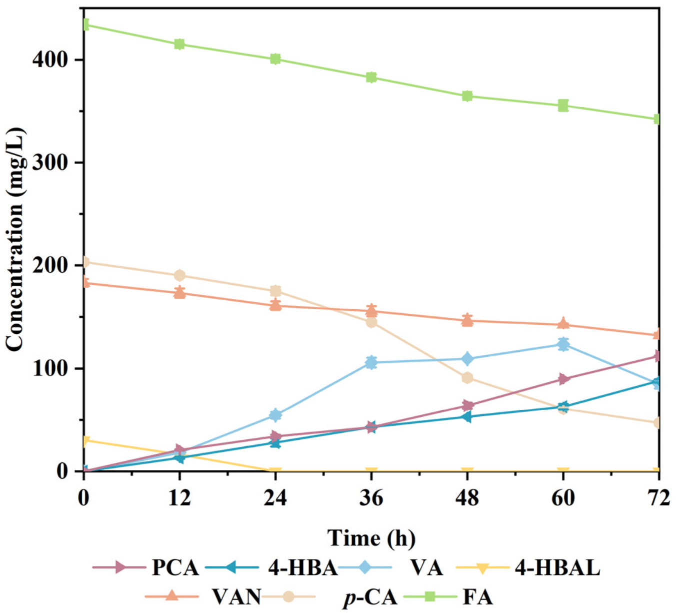

Fig. S6 Production of PCA from 80% (v/v) hydrolysate 2 by KT2

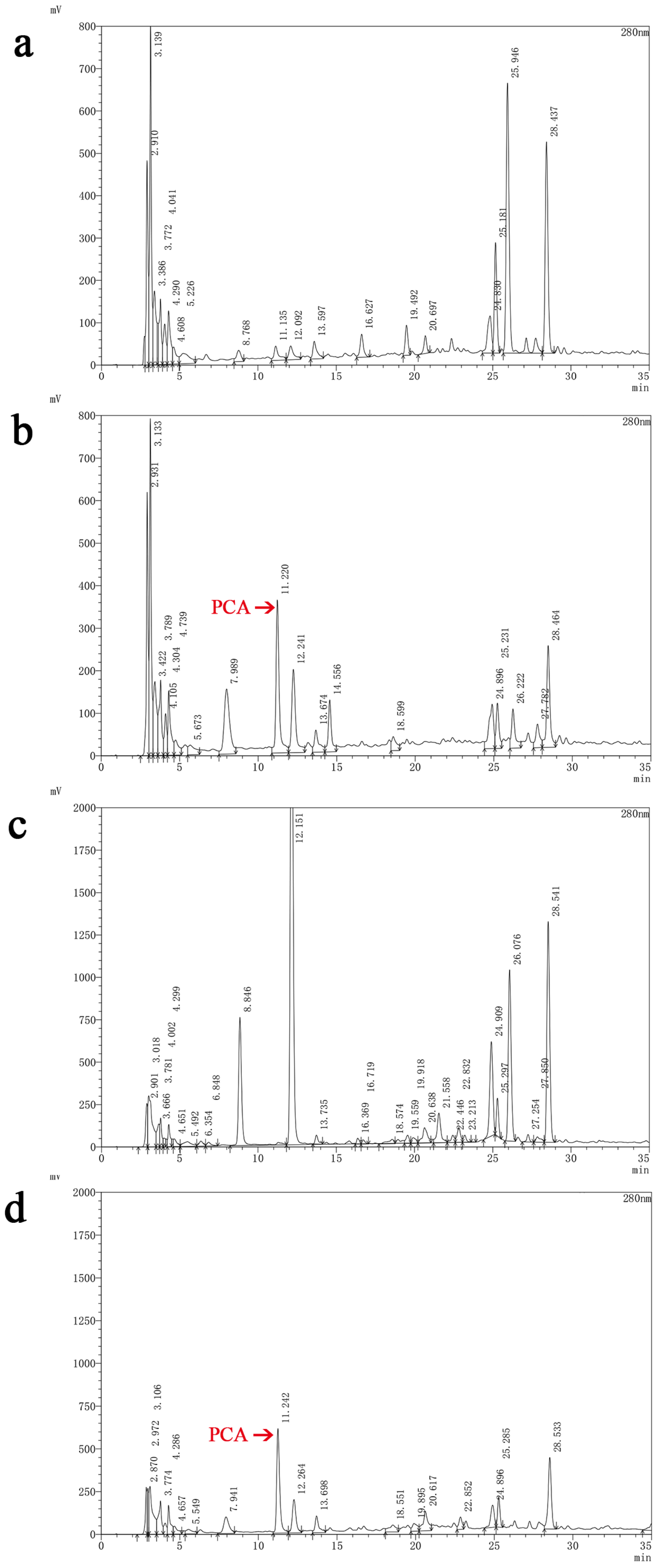

a
b
c
d
mV
280nm
min
PCA
PCA
3.139
2.910
4.041
3.772
3.386
4.290
5.226
4.608
8.768
11.135
12.092
13.597
16.627
19.492
20.697
25.946
28.437
25.181
24.830
3.133
2.931
11.220
3.789
4.739
3.422
4.304
4.105
5.673
7.989
12.241
14.556
13.674
18.599
25.231
28.464
24.896
26.222
27.782
12.151
28.541
26.076
24.909
25.297
27.850
27.254
8.846
4.299
4.002
3.018
3.781
2.901
3.666
4.651
5.492
6.354
6.848
13.735
16.369
16.719
18.574
19.559
19.918
20.638
21.558
22.446
22.832
23.213
3.106
2.972
2.870
4.286
3.774
4.657
5.549
7.941
11.242
12.264
13.698
18.551
19.895
20.617
22.852
24.896
25.285
28.533

Fig. S7 HPLC analysis of PCA production from hydrolysates 1 and 2. (a) 0 h of hydrolysate 1, (b) 72 h of hydrolysate 1, (c) 0 h of hydrolysate 2, (d) 0 h of hydrolysate 2.

Table S1 Primers used in this study, the restriction endonuclease sites are underlined.

Primers	Primer sequence 5'-3'
pcaGH-up-f	gaattcactgaatgaggtgcggccgttc (*Eco*R I)
pcaGH-up-r	cccttacgcctgactacaagacgcgaagtggatgggaagacg
pcaGH-down-f	cgtcttcccatccacttcgcgtcttgtagtcaggcgtaaggg
pcaGH-down-r	ggatccgcgcttgtaccactgggggc (*Bam*H I)
vanAB-f	atttcacacaggaaacagctatgtaccccaaaaacacctg
vanAB-r	gcgaattttaacaaaatattaacgctcagatgtccagcacca
pBBR1MCS-2-f	agctgtttcctgtgtgaaat
pBBR1MCS-2-r	gcgttaatattttgttaaaattcgc
